# Estimating the effective fields of spin configurations using a deep learning technique

**DOI:** 10.1038/s41598-021-02374-0

**Published:** 2021-11-25

**Authors:** D. B. Lee, H. G. Yoon, S. M. Park, J. W. Choi, H. Y. Kwon, C. Won

**Affiliations:** 1 Department of Physics, Kyung Hee University, Seoul, 02447 South Korea; 2 Center for Spintronics, Korea Institute of Science and Technology, Seoul, 02792 South Korea

**Keywords:** Ferromagnetism, Computational science

## Abstract

The properties of complicated magnetic domain structures induced by various spin–spin interactions in magnetic systems have been extensively investigated in recent years. To understand the statistical and dynamic properties of complex magnetic structures, it is crucial to obtain information on the effective field distribution over the structure, which is not directly provided by magnetization. In this study, we use a deep learning technique to estimate the effective fields of spin configurations. We construct a deep neural network and train it with spin configuration datasets generated by Monte Carlo simulation. We show that the trained network can successfully estimate the magnetic effective field even though we do not offer explicit Hamiltonian parameter values. The estimated effective field information is highly applicable; it is utilized to reduce noise, correct defects in the magnetization data, generate spin configurations, estimate external field responses, and interpret experimental images.

## Introduction

Recently, artificial intelligence technology has begun to be applied as an efficient way to study various scientific systems. In condensed matter physics, kernel methods^1^ and simple neural networks have been successfully employed to study various subjects, such as many-body problemss^2,3^ and phase transitions^4–6^. In addition, deep learning techniques have achieved remarkable success in modeling physical data. In particular, it has been intensively applied in the field of magnetism research to investigate various properties of unique magnetic domain structures appearing in low dimensions, estimate Hamiltonian parameters of magnetic complex structures from experimental observation^7–9^ and generate ground states in various systems^10–12^.

Another promising application of deep learning techniques is to reconstruct hidden or unseen information from given data. In the case of colorization models using deep neural networks^13–16^, these models can convert grayscale images to color images based on experience in training; the color is the hidden information in the grayscale images. Similarly, it is expected that these deep learning techniques can be adapted to reveal the hidden information of various scientific data. In magnetism research, several microscopy methods, such as magneto-optical Kerr effect (MOKE) microscopy^17,18^ and scanning transmission X-ray microscopy (STXM)^19–21^, have been developed and utilized to reveal complex magnetic structures by measuring the spatial magnetic moment distribution. However, the effective field, which is a crucial concept for predicting the time evolution and stability of magnetic configurations in micromagnetic simulations, typically cannot be directly measured from microscopy techniques; the effective field can be regarded as hidden information in the spin configuration.

In this study, we use a deep learning technique as a method to obtain the effective field from a given spin configuration. A simulated annealing process implemented by a Monte Carlo method is used to generate our dataset, which is composed of numerous spin configurations formed on a two-dimensional chiral magnetic system^22–24^. We construct a fully convolutional network (FCN), which has been used for several purposes, such as colorization^25,26^ and segmentation^27,28^. The network is trained using the spin configurations in our dataset. We confirm that the trained network can estimate the effective fields properly from given spin configurations. The Hamiltonian of the system is only used in the process of calculating the true effective field of the training dataset and it is not explicitly involved in the network structure. In addition, we introduce a recursive process using the trained network and show that this process can be used for various applications, such as noise reduction, recovering information, estimating effective fields from experimentally observed images, and generating physically plausible states when an external magnetic field is applied.

We show that our deep learning technique successfully estimates the hidden information from the underinformed spin configuration data. Owing to this characteristic, the technique has great potential to be used in analyzing and investigating the physical state by estimating hidden information and hence can be utilized in many other scientific studies.

## Results

### Preliminaries of dataset and training

**Figure 1:**
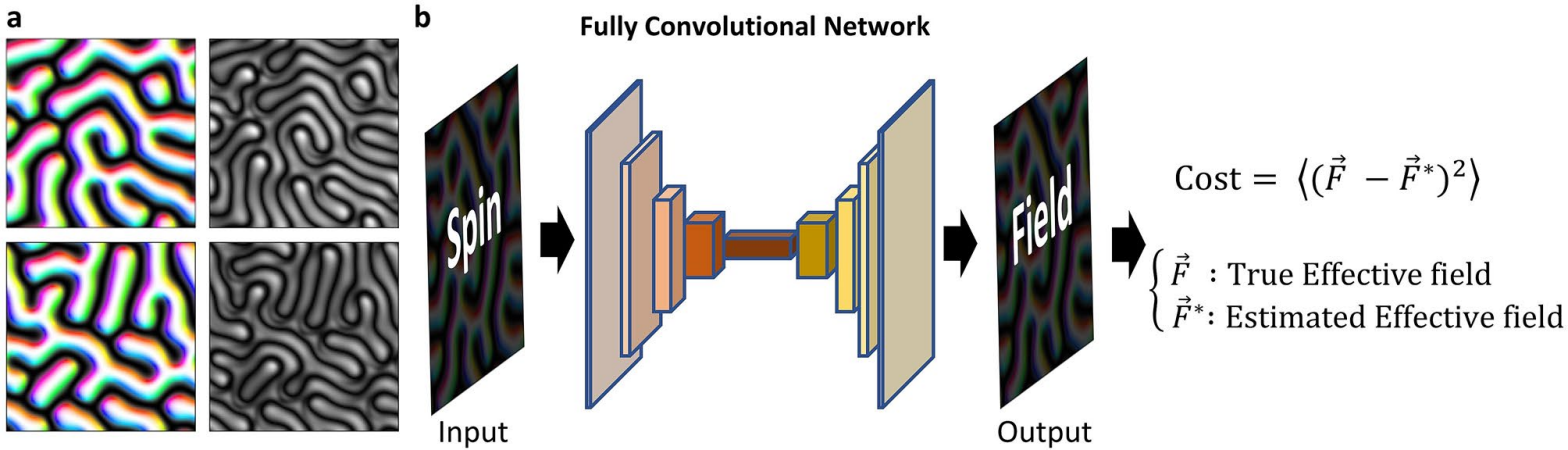
Schematic diagram of training network. (**a**) Samples of labyrinth spin configurations used to train the network and their effective field distributions. Black and white indicate out-of-plane directions, and colors indicate in-plane directions in the spin configurations. The grayscale is used for the intensity of the effective field. (**b**) Schematic of the training process used in this study.

We select magnetization datasets containing several varieties in the spin configuration but with a certain rule that can be learned by our network. For this purpose, the magnetic labyrinth configurations of the two-dimensional magnetic system are used in this study. Magnetic labyrinth configurations, as shown in Fig. 1a, have variety in their shape, but the structures are in their local energy minimum state, and thus, they are energetically and topologically stable. The local magnetic moment is aligned along the effective field. The strength of the magnetic moment in the structure is a constant, whereas the strength of local effective fields varies spatially. The effective field strength is dominantly determined by the exchange interaction, but small spatial variances exist due to the Dzyaloshinskii–Moriya interaction (DMI) and the detailed labyrinth structure. In Fig. 1a, we can see how the effective field strength varies spatially. Therefore, the strength of effective fields is the hidden information that cannot be directly obtained from the spin configurations. In our study, we use a system that includes exchange interactions and DMI, and we train the network to infer the effective field from these two interactions. If our network is applied to a system that has additional energy contributions, such as Zeeman energy or weak anisotropy, their effective field contributions can be added to correct the inferred effective field. If we use a dataset containing additional energies explicitly, it is also possible to train the network to estimate the effective fields from them.

The properties of the labyrinth magnetic structure have been extensively investigated, both numerically and experimentally^22–24,29,30^. With a theoretical model, it is possible to calculate the effective field from the spin structure and to generate a magnetic structure with Monte Carlo simulation. Therefore, the magnetic labyrinth configuration provides a model system for evaluating the trained network and checking whether the network can estimate the physically plausible effective field from the structures. Details of dataset generation are explained in the “Methods” section.

In this study, we use an FCN to estimate the effective field from the spin configuration. Figure 1b shows the schematic network training workflow. We feed the spin configurations from the simulation as the input, and the FCN is trained to estimate the effective field. An FCN can derive output from the input image of any size, even if it is trained with data of a specific size. Thus, we use the FCN as our network, and it can be applied to estimate the effective field from the spin configuration with data of any size. Due to these properties of the FCN, we can apply our network to the magnetization image from experiments as well as data generated by simulation annealing. Details of the network structure are discussed in the “Methods” section.

### Characteristics of the trained network

**Figure 2:**
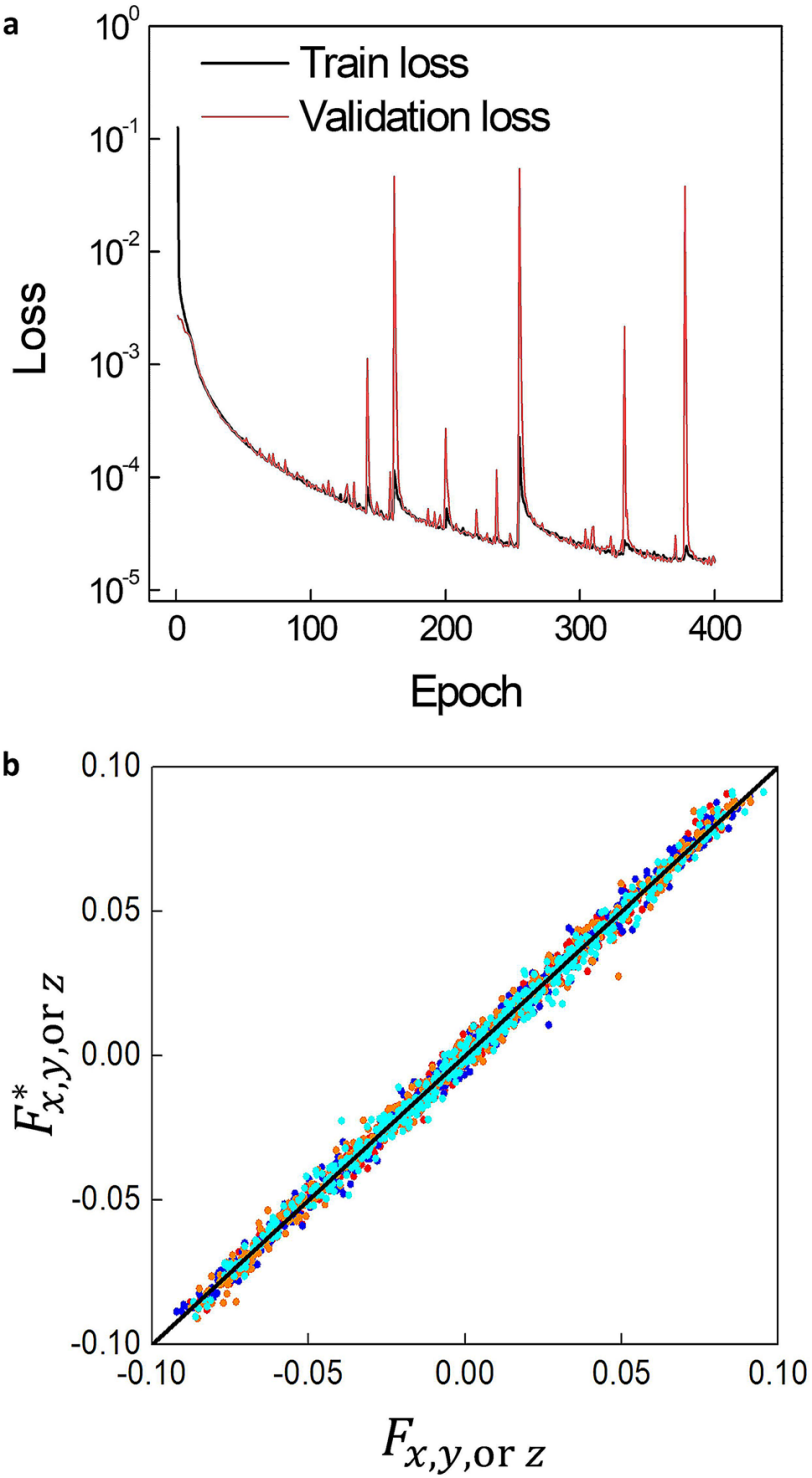
Training, validation, and testing of the network. (**a**) During the network training process, the training loss and validation loss values are calculated at each iteration. The black and red lines indicate the training loss and validation loss, respectively. (**b**) The estimated effective field (${\varvec{F}}_{{{\varvec{x}},{\varvec{y}},\;{\mathbf{or}}\;{\varvec{z}}}}^{{*}}$) from the samples’ test dataset is plotted as a function of the true effective field (${\varvec{F}}_{{{\varvec{x}},{\varvec{y}},\;{\mathbf{or}}\;{\varvec{z}}}}$). The black line shows ${\varvec{F}}_{{{\varvec{x}},{\varvec{y}},\;{\mathbf{or}}\;{\varvec{z}}}} = \varvec{F}_{{{\varvec{x}},{\varvec{y}},\;{\mathbf{or}}\;{\varvec{z}}}}^{{*}}$. The portion in the case of uniform magnetization is subtracted in the graph.

During the network training process, the training loss and validation loss are decreased to the order of $10^{ - 5}$ (Fig. 2a). We first investigate the training results of the deep learning algorithms. This is done by estimating the effective fields from the spin configurations in four randomly chosen samples from the test dataset and analyzing the ratio between the true effective fields $F_{{x,y,\;{\text{or}}\;z}}$ and estimated effective fields $F_{{x,y,\;{\text{or}}\;z}}^{*}$, with the subscript denoting the *x*, *y*, or *z* components of the fields. In Fig. 2b, we see that $F_{{x,y,\;{\text{or}}\;z}}$ and $F_{{x,y,\;{\text{or}}\;z}}^{*}$ have a strong linear correlation.

**Figure 3:**
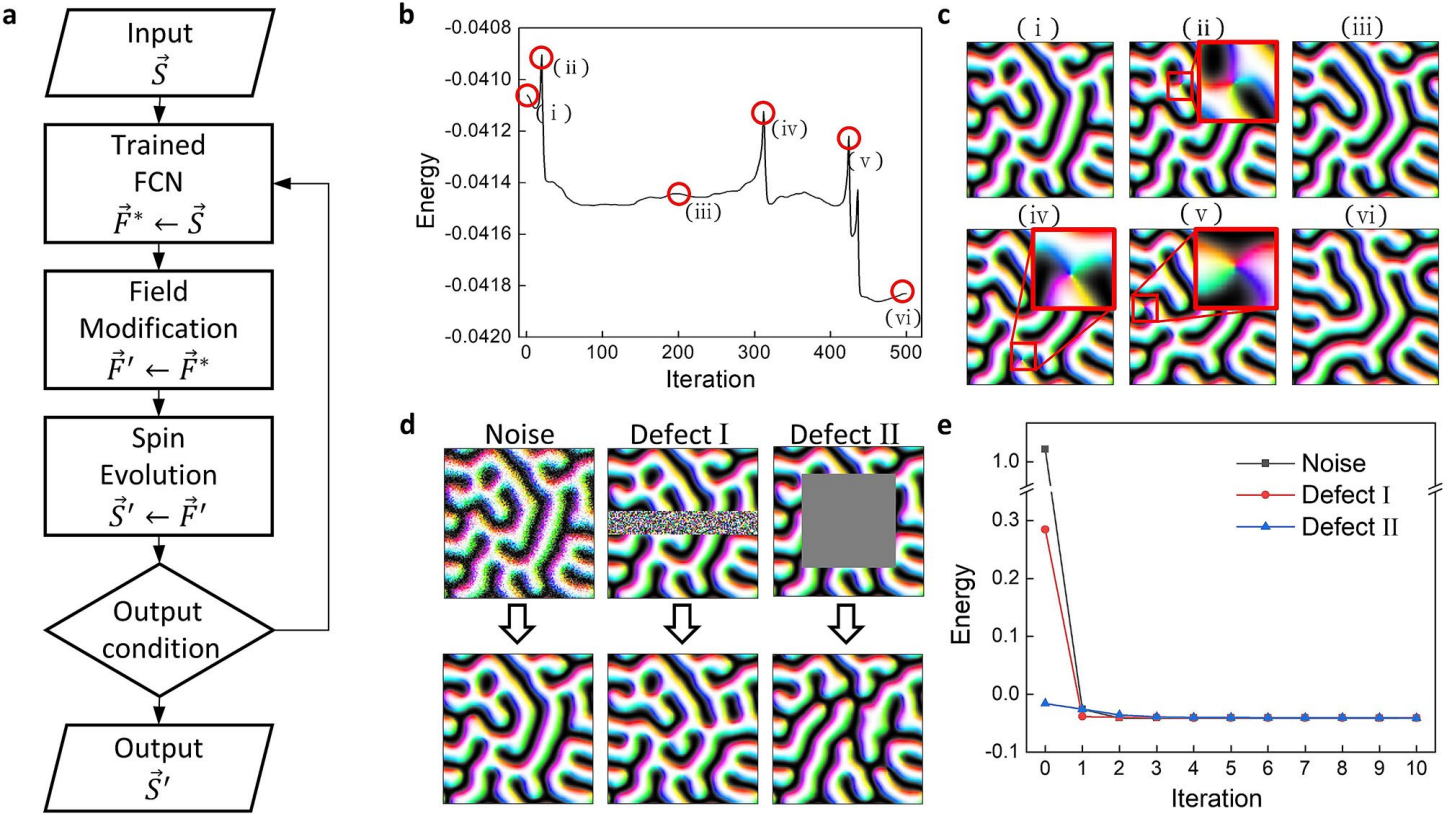
Application of the recursive process. (**a**) A schematic diagram shows the recursive process. (**b** and **c**) An energy graph of the spin configuration during the simple recursive process and images of the spin configuration corresponding to (i–vi) are shown. Zoomed-in images show the nodal points in each image. (**d** and **e**) The noisy and defective spin configurations and the results after iteration of the simple recursive process 10 times. The graph shows the energy change during 10 iterations.

The effective field information obtained from the trained network provides expanded information on the spin structure, and it can be manipulated to recover or evolve the spin structure. When a new spin structure is obtained from the effective field, the trained network can be used to infer its effective field again. To apply the effective field information, we use the recursive process presented in Fig. 3a. The recursive process is composed of feeding the input spin configuration to the trained network, generating a new spin configuration from the output effective field, and refeeding the spin configuration as a new input of the network. First, the trained network provides the estimation of the effective field, and then, the effective field is modified according to the application necessity. Additional fields such as external fields or fluctuations, which are not considered in the training dataset, can be included in the estimated effective field. With effective field information, a new magnetization map is generated by an evolutionary method. In a statistical study, magnetic moments can be sampled by thermal distribution. In a dynamic study, they can be evolved using equations of motion such as the Landau–Lifshitz–Gilbert (LLG) equation so that they precess around the effective field inferred by our network. In our discussion, we use a spin evolution method where the magnetic moments are immediately adjusted to be parallel with the effective field in a step (greedy method) because it is the simplest method for evaluating our network. Details of the recursive process are explained in the “Methods” section.

Figure 3b shows how the magnetic energy evolves with the simple recursive process in which no field modification is used and the greedy method is applied to the magnetic moments. Energy is calculated through the dot product of the spin vector and the effective field. When the energy calculated from the true effective field is compared with the energy calculated from the estimated effective field, the accuracy is approximately 99.95%. Although the network is only trained to estimate the effective field from the spin configuration, we find that the initial spin configuration can evolve to a lower energy state during the recursive process. Some truncated magnetic structures are connected, and some connected structures are separated during a recursive process, resulting in the total energy being lowered. Although there are topological energy barriers indicated by the energy peaks in Fig. 3c, transitions among metastable states appear in the recursive process.

The reorganization of the magnetic structure is a notable result. In general, changing the topological structure requires a significant amount of energy, as each metastable state is located at a local energy minimum. Thus, considering that the training is only performed with thermal-fluctuation-less structures, it is interesting that escaping the local minimum state naturally occurs in the FCN's recursive process. We speculate that the reason for this phenomenon is because we train to estimate the effective field from various metastable spin configurations, and the estimated value is not completely accurate. So, it can reflect the general feature of the group of metastable states. Therefore, spin configuration can be changed to another plausible state during the recursive process, passing energy barriers among metastable states. In case that we apply our network to the spin systems without global stable states due to frustrations, we expect that various metastable states can be searched during the recursive process.

The initial attempts at the recursive process show that the network suitably learns the general properties of the spin configurations in the training. It tends to remove atypical features in the spin configurations and fix them to have general features learned in the training. These characteristics enable us to apply the network to correct or modify spin configurations. In the following sections, we show several application methods, which fully exemplify the advantages of these aforementioned characteristics.

### Application: noise removal and defect correction

One possible network application is denoising, a field in which artificial intelligence is efficiently utilized^31,32^. To see if our network can be effective for this purpose, we intentionally injected random noise and defects in the spin configuration into our datasets. Random noise was injected into the spin configurations using $\hat{S}^{\prime } = L_{2} \left( {\hat{S} + \alpha \hat{R}} \right)$, where $\hat{S}^{\prime }$ and $\hat{S}$ are the noisy and noiseless spin configurations, respectively $\hat{R}$ is a unit vector map randomly oriented in any direction, $\alpha$ is the coefficient for varying the amplitude of the random map, and $L_{2}$ is the L2-normalization process. The representative case of $\alpha = 2.5$ is shown in the leftmost column of Fig. 3d. When we feed the noisy spin configuration into the trained network, the noise is almost instantly removed within a few iterations; the energy decrease indicates that the noise has been removed.

We also intentionally place defect sites in the spin configuration dataset. The process of injecting defects involves erasing the magnetization information in a specific region of the spin configuration. We use two types of defects: Defect I is made by erasing the middle rows of the data and adding random unit vectors in the erased part (center column of Fig. 3d), whereas Defect II is made by simply erasing a square-shaped center region of the data (rightmost column of Fig. 3d). When we feed the defect-containing-spin configuration into the trained network, the defect regions in the spin configurations are reconstructed such that they show plausible spin configurations.

The recursive process of our trained network also lowers the spin configuration energy, as shown in Fig. 3e. The energy decrease is achieved by removing noise and reconstructing defects. From these results, we clearly observe that the trained network is capable of outputting plausible effective fields that are used to construct a spin configuration even when the input magnetization map does not contain complete information. The output result is built to have lower energy and hence becomes one of the most plausible states based on the training set information.

### Application: extraction of hidden information from experimental data

**Figure 4:**
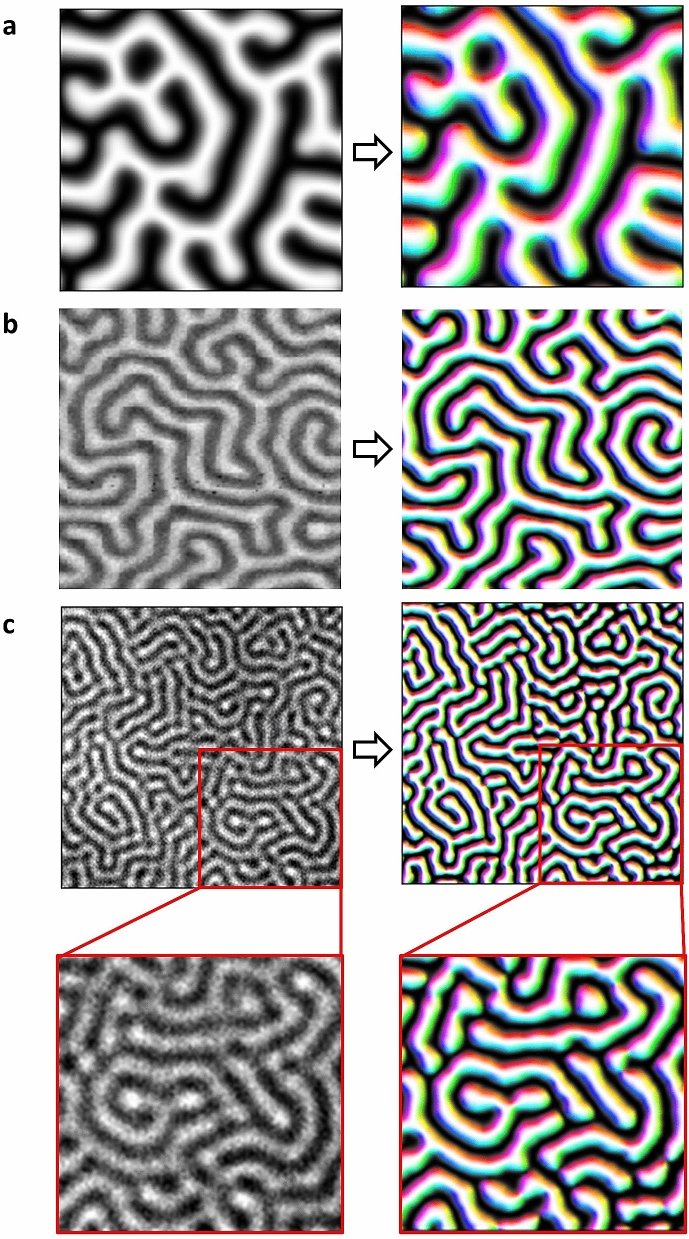
Estimating the *x*, *y*, and *z* components of the effective field from the test and experimental data. (**a**) The estimated effective field when feeding simulated test data, which contains only the *z*-axis component, to the trained network. (**b**) The estimated effective field result of feeding the STXM data to the trained network. (**c**) The estimated effective field result of feeding the MOKE microscopy data to the trained network. The figures on the left have only one axial spin component as input data, and the figures on the right are the estimated result by the trained network.

Given that the trained network has the characteristic of estimating the effective field from the spin configuration without full information, we feed simulated test data that contain only one magnetization vector information component. In Fig. 4a, we see that the network successfully estimates all components (*x*, *y*, and *z*) of the effective field even when the input data contain only one (*z*) spin configuration component.

This capability of our network is fully exhibited when applied to experimental data. Most magnetic microscopy techniques, such as STXM and MOKE microscopy, only provide one axial spin component; thus, it is necessary to infer other directional components from it. To prove the capability, our network is applied to actual experimental data where only one magnetization component is measured. Figure 4b and c shows the results when the network input data are experimental magnetic domain images of a [Pt(3 nm)/GdFeCo(5 nm)/MgO(1 nm)]_20_ multilayer system. Detailed information about the experimental environment is given in a previous study^33^. The magnetic domains shown in Fig. 4b and c are observed using STXM and MOKE, respectively.

We note that the effective field or the in-plane magnetization inferred by our network is valid if the Hamiltonian used for the training data is applicable to the experimental system. Therefore, the method can be suitably applied in cases where the certain theoretical model is known but the experimental data do not provide the entire information. In our case, all three components of the effective fields are well estimated by the network. Although the experimental data are unnormalized, the image size is different from the training data, and only one axial spin component is given. The Hamiltonian used in our training includes the interfacial DMI, typical of Pt/GdFeCo/MgO multilayers^33^. As a result, we see in-plane components from the effective field (Fig. 4b, c), as we expect in the systems where the interfacial DMI induces Neél-type domain walls.

### Application: generative model

**Figure 5:**
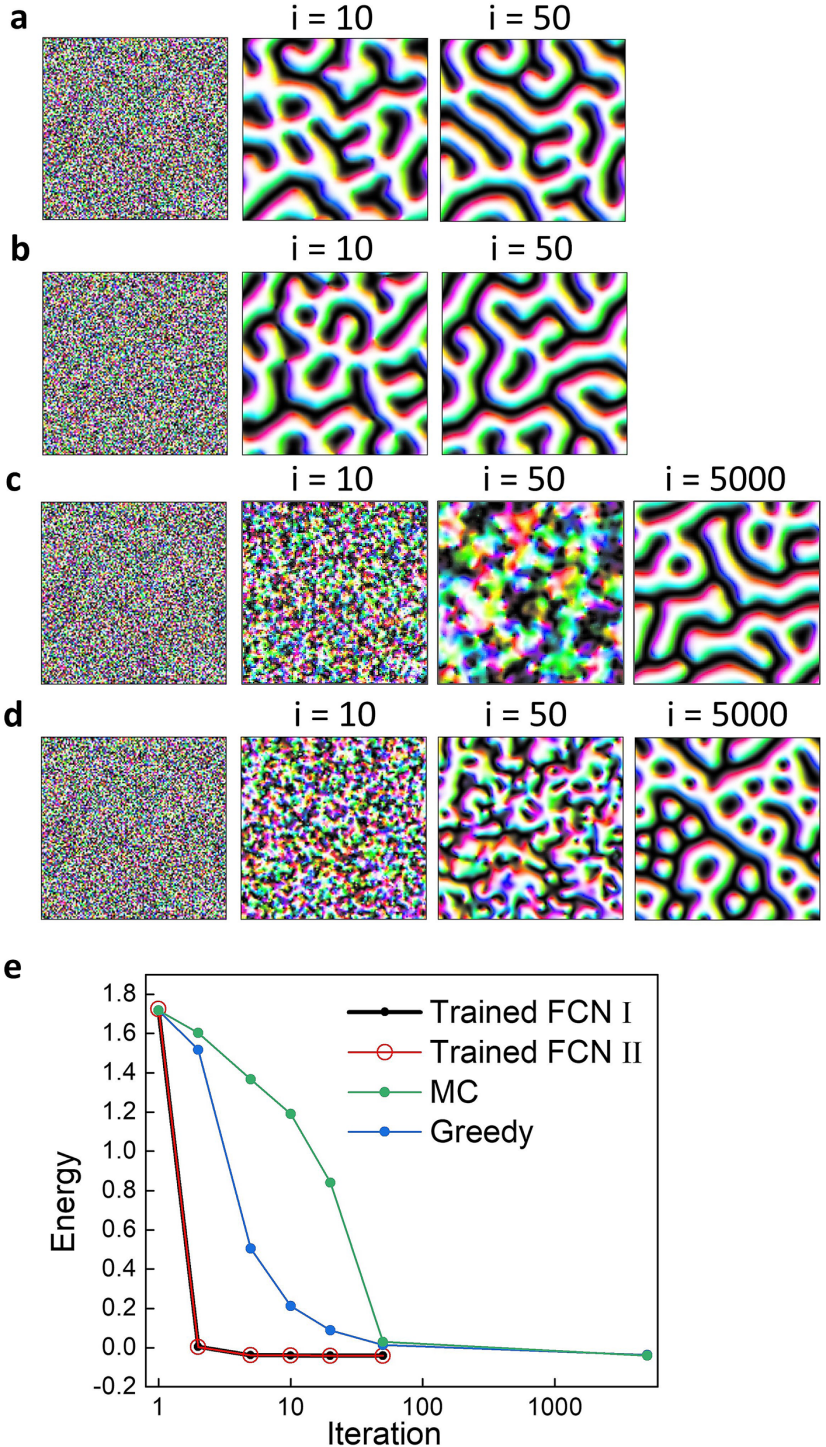
Data generation of the trained network, MC, and greedy algorithm. (**a**) Spin configuration data are generated during 50 iterations by feeding a random map to the trained network. (**b**) Another random map is fed to the trained network and shows the result of 50 iterations. (**c**, **d**) The results show the data generated after 5000 iterations using the MC method and greedy algorithm. Each Hamiltonian parameter used the same data used for training. (**e**) Energy changes during iterations are shown for each method. The black and red data points represent data generation from different random maps of the trained network. The green and blue points indicate the MC method and greedy algorithm.

The trained network in this study also has the potential to generate new spin configurations as a generative deep learning model, as shown in Fig. 5a. Details of the generation recursive process are given in the “Methods” section. When we feed a random spin map to the network, the output data become a plausible spin configuration within a few recursive iterations. Figure 5b shows that if we feed a different random map to the network, another spin configuration is output. From these results, the trained network can be considered a generative model that generates the different spin configurations whenever different random maps are seeded.

We compare spin configurations generated by several generative methods: the Monte Carlo (MC) method, the greedy method, and spin configurations generated by our trained network. In the MC method, we generate the spin configuration by dropping the temperature from higher than the Curie temperature to zero. In Fig. 5c, the spin configuration, such as the ones we used to train the network, is only generated after thousands of iterations when using the MC method. The greedy method is a model that generates spin configuration with the MC method in a state where the temperature is 0. In the greedy method, the result of the final iteration (Fig. 5d) shows multiple skyrmions. Not only is the physical result different in this case, but the energy of this multiskyrmion state is higher than the data of the spin configuration dataset that we used to train the network.

To quantitatively investigate whether the spin configurations generated by a recursive process are physically plausible, we compare the energy of the resultant states of this process with those of the greedy and MC methods (Fig. 5e). The energy values from the recursive process are minimized in a few iterations. In contrast, the greedy and MC methods require thousands of iterations to generate a sufficiently minimized energy state. This clearly shows that the generation method using the recursive process proposed in this study can generate a new metastable spin configuration with a much lower computational cost. Additionally, since we use the FCN, we can generate a new spin configuration of any size by feeding a random map of the desirable size other than $128 \times 128$ (size used for training) into the network. This again exemplifies the advantage of our network as a spin configuration generator.

### Application: addition of external fields

Experimentally, it is well known that when an appropriate out-of-plane (*z*-direction) external field is applied, the labyrinth spin configuration changes to magnetic skyrmions before all magnetic moments become uniformly aligned when the out-of-plane field is further increased^34–37^. Since the trained FCN estimates the effective field without any external field, we can include additional fields in the recursive process to observe how the additional external field modifies the original structure.

**Figure 6:**
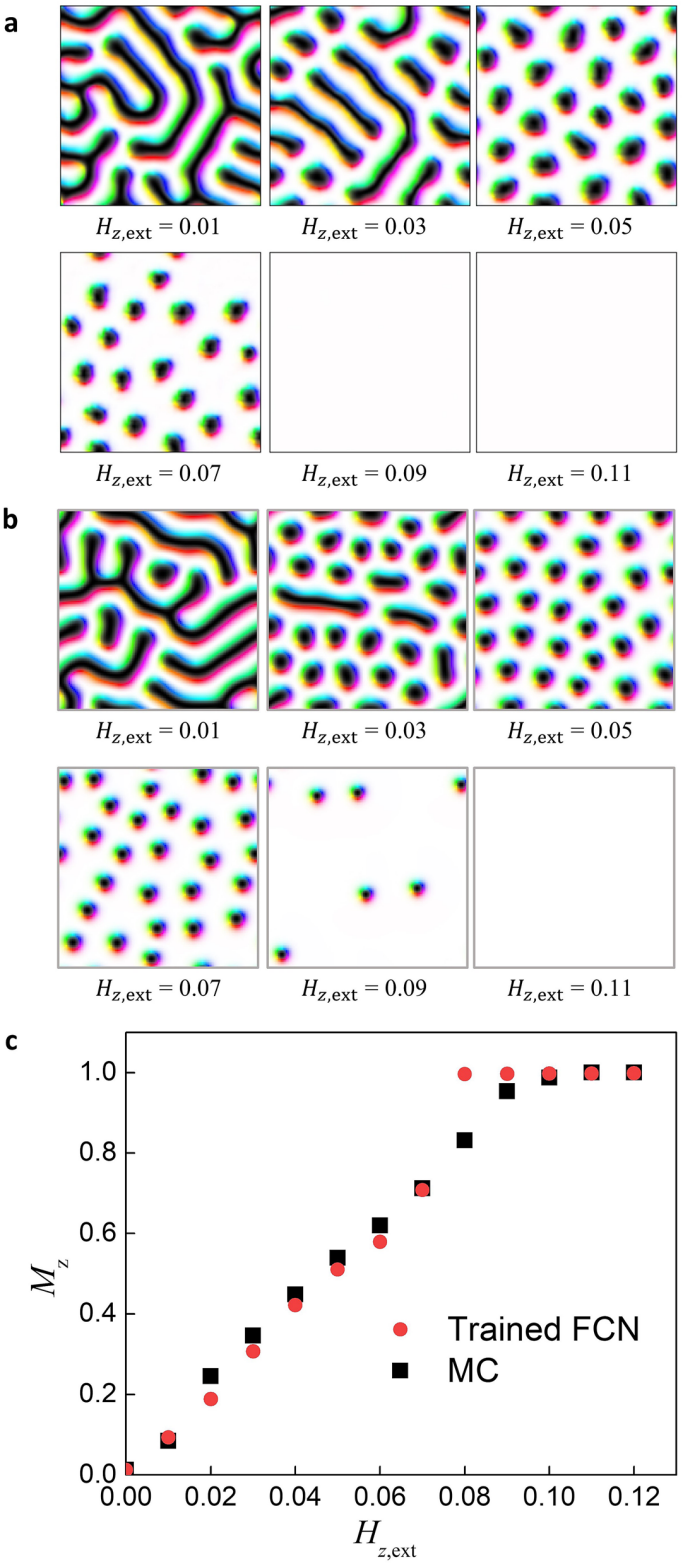
Field-dependent spin configuration images and magnetization using our trained network and MC method using a recursive process. (**a**) The spin configuration resulting from our trained network using the recursive process with an out-of-plane (*z*-direction) external field. (**b**) The spin configuration resulting from the MC method using the recursive process with an out-of-plane (*z*-direction) external field. (**c**) ${\varvec{M}}_{{\varvec{z}}}$ vs. ${\varvec{H}}_{{{\varvec{z}},{\mathbf{ext}}}}$ graph, in which the red and black dots indicate the trained network and MC method.

We add the external field in the field modification step in the recursive process such that the total effective field to produce the magnetization map in the next iteration becomes $\vec{F}^{\prime } = \vec{F}^{*} + H\hat{z}$. The other type of effective field, such as the anisotropy field for weak anisotropy energy or the Langevin field for thermal fluctuation, can be added similarly when necessary. Details of the field addition in the recursive process are given in the “Methods” section. We use a labyrinth spin configuration as the initial state. As shown in Fig. 6a, the labyrinth structure starts to break into smaller domains when a field of $H_{{z,{\text{ext}}}} = 0.03$ is applied. At $H_{{z,{\text{ ext}}}} = 0.05$, the skyrmion spin configurations appear. When the field is further increased, the skyrmion configuration gradually disappears ($H_{{z,{\text{ ext}}}} = 0.07$) and becomes gradually saturated out-of-plane ($H_{{z,{\text{ ext}}}} = 0.09$). To confirm that these results are reasonable, we similarly apply the external field with the MC method (Fig. 6b). In the MC method, we find the spin configuration as the temperature is decreased from above the Curie temperature to zero while applying an external field. The results of applying the field to the trained FCN and the MC method are interestingly similar. Figure 6c shows the magnetization as a function of the external field. We see that two graphs from our FCN and MC methods show almost identical field-dependent magnetizations. During the training process, the network is trained only to estimate the effective field without any external field; we confirm that adding external fields to our method can generate physically plausible states.

## Conclusion

We devised a novel method based on a deep learning technique to estimate the effective field information of spin configurations. An FCN was trained using various spin configurations generated by a simulated annealing process. We confirmed that the trained network can estimate the effective fields of input spin configurations even though we did not offer the explicit Hamiltonian parameters that are used in the data generation process.

Through the recursive process introduced in this study, we found a surprising feature of the trained network: it prefers to make the output spin configurations more stable or more plausible than the input spin configurations. We utilized useful features to devise several application methods for various purposes, such as noise reduction, correcting defects, estimating external field responses, and inferring the hidden information of underinformed experimental data. Generating plausible spin configurations with a less computational cost is also a possible application of the trained network, as presented in this study. We believe that the interesting properties and various applicability of our method can be adopted as novel numerical methods in many other scientific research areas.

## Methods

### Dataset generation

The dataset is chosen to evaluate whether the network structure can properly estimate the effective fields from the spin configurations. The input data should be well characterized under certain conditions, while they should have a variety of structures. Therefore, in this study, we generate magnetic labyrinth configurations as a dataset. They have been extensively studied in two-dimensional magnetic systems due to the potential for new spin device applications, and it is well known that a phase transition to a skyrmion structure occurs with an external field. These properties provide advantages for evaluating our network.

To implement two-dimensional magnetic systems, we use the Heisenberg spin model in a square lattice system of $128 \times 128{ }$ size. The magnetic labyrinth configurations are generated under the Hamiltonian shown in Eq. (1),

1 $${\mathcal{H}} = - J\mathop \sum \limits_{{\left\langle {ij} \right\rangle }} \vec{S}_{i} \cdot \vec{S}_{j} + \mathop \sum \limits_{{\left\langle {ij} \right\rangle }} \vec{D}_{ij} \cdot \left( {\vec{S}_{i} \times \vec{S}_{j} } \right),$$

where $\vec{S}$ is a normalized spin vector, $J$ is an exchange parameter, and $\vec{D}_{ij}$ is a DMI vector. $i$ and $j$ represent the spin sites index, and the summation is on every nearest pair site. The ratio between $J$ and $|\vec{D}_{ij} |$ determines the length scale of the magnetic structure, and we choose it at $J{/}|\vec{D}_{ij} | = 1{/}0.3$ to have enough structure in a simulation size. The effective field of the spin configuration is also obtained from Eq. (1), $\vec{F} = - \vec{\nabla }_{{\vec{S}}} {\mathcal{H}}$.

A simulated annealing process is used to generate various labyrinth spin configurations; the temperature of the system is gradually decreased from above the Curie temperature to zero temperature. The total number of generated data points is 30,100, and we divide it into three subdatasets: training, validation, and test. These three datasets are composed of 25,000, 5000, and 100 spin configurations, respectively.

### Network structure and loss function

The goal of this study is to devise an algorithm for estimating the effective fields from the spin configurations using the deep learning technique. We construct a neural network structure to obtain the effective field from the input spin configuration. The structure is similar to an autoencoder that has an encoder and a decoder. The encoder, composed of four FCN layers with 8, 16, 32, and 64 filters, abstracts the spin configuration. The filter sizes are $3 \times 3$. Since our spin configuration dataset is generated under the periodic boundary condition, we add a periodic padding process in front of all FCN layers to train with the same conditions. After every FCN layer in the encoder, we attach the batch normalization layer, rectified linear unit (ReLU) activation, and max-pooling layer whose pooling size is $3 \times 3$. The decoder decodes the abstracted information into the effective field. It is constructed using four upsampling blocks, and the single block is composed of both an upsampling layer with a $2 \times 2$ filter and an FCN layer with a $3 \times 3$ filter. The number of filters for the FCN layers in upsampling blocks are 32, 16, 8, and 3 for each. After the decoder, we add one more FCN layer, the last FCN layer, with three filters. The periodic padding process we use in the encoder is added in front of all FCN layers in the decoder. The batch normalization layer and ReLU activation are attached after all FCN layers in the decoder except for the last FCN layer. The input and output data dimensions are the same as [400, 128, 128, 3]; the input data are hundreds of spin configurations generated under the Hamiltonian shown in Eq. (1), and the output data are hundreds of two-dimensional vector maps composed of three-dimensional vectors.

We want to train our network structure to make the output vector maps become the effective fields of input spin configurations. Therefore, the mean squared error (MSE) $\left( {\vec{F} - \vec{F}^{*} } \right)^{2}$ is used as a loss function, where $\vec{F}$ is a true effective field and $\vec{F}^{*}$ is the estimated effective field. The difference between the true effective fields of input spin configurations and the output vector maps is used as the total loss of our network, which should be minimized during the training process. The minimization of the total loss means that the output vector maps become identical to the true effective input data fields; thus, after the training process, our network structure can appropriately estimate the effective input data fields. The Adam optimizer is adopted to minimize the total loss with a 0.01 learning rate.

### Recursive process

In the recursive process shown in Fig. 2a, the spin configuration is fed as input data, and the trained FCN estimates the effective field. Through the field modification step, we can change the field in the way we want. In most discussions, we do not modify the field $\left( {\vec{F}^{\prime } \leftarrow \vec{F}^{*} } \right)$, but in the last discussion on the effect of the external field, we add a constant field to the field from the FCN $\left( {\vec{F}^{\prime } \leftarrow \vec{F}^{*} + H\hat{z}} \right)$. The effective field is used to change the spin configuration in the spin evolution step. Suitable methods can be applied depending on the purpose. In our study, we simply align the spin direction parallel to the effective field $\left( {\vec{S}^{\prime } \leftarrow \vec{F}^{\prime } {/}|\vec{F}^{\prime } |} \right)$. This process is repeated until the output condition is satisfied.

## References

[1] Xue D (2016). Accelerated search for materials with targeted properties by adaptive design. Nat. Commun..

[2] Carleo G, Troyer M (2017). Solving the quantum many-body problem with artificial neural networks. Science.

[3] Cai Z, Liu J (2018). Approximating quantum many-body wave functions using artificial neural networks. Phys. Rev. B.

[4] Wang L (2016). Discovering phase transitions with unsupervised learning. Phys. Rev. B.

[5] van Nieuwenburg EPL, Liu YH, Huber SD (2017). Learning phase transitions by confusion. Nat. Phys..

[6] Rem BS (2019). Identifying quantum phase transitions using artificial neural networks on experimental data. Nat. Phys..

[7] Wang D (2020). Machine learning magnetic parameters from spin configurations. Adv. Sci..

[8] Kwon HY (2020). Magnetic Hamiltonian parameter estimation using deep learning techniques. Sci. Adv..

[9] Singh VK, Han JH (2019). Application of machine learning to two-dimensional Dzyaloshinskii–Moriya ferromagnets. Phys. Rev. B.

[10] Kwon HY, Kim NJ, Lee CK, Won C (2019). Searching magnetic states using an unsupervised machine learning algorithm with the Heisenberg model. Phys. Rev. B.

[11] Kwon HY (2019). An innovative magnetic state generator using machine learning techniques. Sci. Rep..

[12] Kwon HY (2021). Magnetic state generation using Hamiltonian guided variational autoencoder with spin structure stabilization. Adv. Sci..

[13] Limmer, M. & Lensch, H. P. A. Infrared colorization using deep convolutional neural networks. In *15th IEEE International Conference on Machine Learning and Applications* 61–68 (IEEE, 2016).

[14] Cheng, Z., Jiao, S., Yang, Q. & Sheng, B. Deep colorization. In *Proceeding of the IEEE International Conference on Computer Vision* 415–423 (2015).

[15] Zhang, R., Isola, P. & Efros, A. A. Colorful image colorization. In *European Conference on Computer Vision* 649–666 (2016).

[16] Poterek Q, Herrault PA, Skupinski G, Sheeren D (2020). Deep learning for automatic colorization of legacy grayscale aerial photographs. IEEE J. Sel. Top. Appl. Earth Obs. Remote Sens..

[17] Allwood DA, Xiong G, Cooke MD, Cowburn RP (2003). Magneto-optical Kerr effect analysis of magnetic nanostructures. J. Phys. D Appl. Phys..

[18] Rave W, Schäfer R, Hubert A (1987). Quantitative observation of magnetic domains with the magneto-optical Kerr effect. J. Magn. Magn. Mater..

[19] Fischer P (1998). Imaging of magnetic domains by transmission X-ray microscopy. J. Phys. D Appl. Phys..

[20] Ono K (2011). Element-specific magnetic domain imaging of (Nd, Dy)-Fe-B sintered magnets using scanning transmission X-ray microscopy. IEEE Trans. Magn..

[21] Bykova I (2018). Soft X-ray ptychography for imaging of magnetic domains and skyrmions in sub-100 nm scales. Microsc. Microanal..

[22] Kwon HY (2010). A study of the stripe domain phase at the spin reorientation transition of two-dimensional magnetic system. J. Magn. Magn. Mater..

[23] Kwon HY, Won C (2014). Effects of Dzyaloshinskii–Moriya interaction on magnetic stripe domains. J. Magn. Magn. Mater..

[24] Kwon HY, Kang SP, Wu YZ, Won C (2013). Magnetic vortex generated by Dzyaloshinskii–Moriya interaction. J. Appl. Phys..

[25] Lin J (2018). Automatic colorization using fully convolutional networks. J. Electron. Imaging.

[26] Kim HK, Yoo KY, Park JH, Jung HY (2019). Asymmetric encoder–decoder structured FCN based LiDAR to color image generation. Sensors.

[27] Long, J., Shelhamer, E. & Darrell, T. Fully convolutional networks for semantic segmentation. In *Proceedings of the IEEE Conference on Computer Vision and Pattern Recognition* 3431–3440 (2015). 10.1109/TPAMI.2016.2572683 27244717

[28] Xing Y, Zhong L, Zhong X (2020). An encoder–decoder network based FCN architecture for semantic segmentation. Wirel. Commun. Mob. Comput..

[29] Moon KW (2019). Measuring the magnetization from the image of the stripe magnetic domain. Phys. Rev. A.

[30] Agrawal P, Büttner F, Lemesh I, Schlotter S, Beach GSD (2019). Measurement of interfacial Dzyaloshinskii–Moriya interaction from static domain imaging. Phys. Rev. B.

[31] Tian C (2020). Deep learning on image denoising: An overview. Neural Netw..

[32] Xie J, Xu L, Chen E (2012). Image denoising and inpainting with deep neural networks. Adv. Neural Inf. Process. Syst..

[33] Woo S (2018). Current-driven dynamics and inhibition of the skyrmion Hall effect of ferrimagnetic skyrmions in GdFeCo films. Nat. Commun..

[34] Peng L (2018). Relaxation dynamics of zero-field skyrmions over a wide temperature range. Nano Lett..

[35] Yu X (2018). Aggregation and collapse dynamics of skyrmions in a non-equilibrium state. Nat. Phys..

[36] Wild J (2017). Entropy-limited topological protection of skyrmions. Sci. Adv..

[37] Westover AS, Chesnel K, Hatch K, Salter P, Hellwig O (2016). Enhancement of magnetic domain topologies in Co/Pt thin films by fine tuning the magnetic field path throughout the hysteresis loop. J. Magn. Magn. Mater..

